# Xanthogranulomatous osteomyelitis

**DOI:** 10.1007/s10195-011-0165-8

**Published:** 2011-11-11

**Authors:** Abolhasan Borjian, Farshid Rezaei, Mohammad Amin Eshaghi, Hamidreza Shemshaki

**Affiliations:** 1 Department of Orthopedic Surgery, Isfahan University of Medical Sciences, Isfahan, Iran; 2 Department of Infectious Disease, Isfahan University of Medical Sciences, Isfahan, Iran; 3 Research Medical Center, Kerman University of Medical Sciences, Blv Jahad, Kerman, Iran

**Keywords:** Xanthogranulomatous osteomyelitis, Inflammatory, Humerus, Fibula

## Abstract

Xanthogranulomatous osteomyelitis is a rare type of inflammatory process which is characterized by composition of immune cell aggregation on histological studies. Delayed-type hypersensitivity reaction of cell-mediated immunity may be implicated in its pathogenesis. Gross and radiological examination can mimic malignancy, and differentiation should be confirmed by histopathological evaluation. We describe the case of a 14-year-old Afghan boy presenting with pain in right shoulder and left leg with prior history of trauma. Fever, limitation in right shoulder range of motion, and tenderness in right shoulder and left thigh were detected following examination. Mild leukocytosis, elevated alkaline phosphatase, and increased erythrocyte sedimentation rate with negative C-reactive protein (CRP) were revealed. X-ray imaging showed mixed density, periosteal reaction, and cortical disruption. Computed tomography (CT) scan revealed lesions involving medulla and cortex, periosteal reaction with soft tissue component, and bone marrow infiltration in right humerus and left fibula. On magnetic resonance imaging (MRI), signal abnormalities in medulla, metaphysis, and diaphysis of the left fibula associated with cortical irregularity and diffuse soft tissue hypersignal areas were demonstrated. Finally, xanthogranulomatous osteomyelitis was confirmed by histological sample. The clinical manifestations and radiographic and laboratory findings of this rare condition are discussed.

## Introduction

Xanthogranulomatous inflammation process, including xanthogranulomatous osteomyelitis (XO), is a chronic inflammatory disease characterized histologically by abundant foamy periodic acid-Schiff (PAS)-positive histiocytes in the initial stages, giant cells, fibrosis, and calcification together with polymorphonuclear leukocytes, activated plasma cells, and lymphocytes of polyclonal origin. It can involve any organ, but the most common sites are kidney and gallbladder [1, 2]. Other organs such as lung, brain, and bone are rarely affected. Bone involvement is accompanied by systemic and regional clinical presentations such as pain, fever, and leukocytosis. Delayed-type hypersensitivity reaction of cell-mediated immunity may be implicated in its pathogenesis. Grossly, it is a mass-like lesion extending to enclosing tissues which can mimic infiltrative cancer [3]. There is a long list of differential diagnoses, with the main ones discussed being Langerhans cell histiocytoses, Erdheim–Chester disease (ECD), chronic recurrent multifocal osteomyelitis (CRMO), and metastatic renal cell carcinoma. Clinical background and histopathological examination differentiate between them for exact diagnosis.

There are only four reported cases of xanthogranulomatous osteomyelitis in previous literature: two in 1984 [4], one in 2007 [5], and one in 2009 [6].

In this report we describe an entity involving two bones independently, one in upper and the other in lower extremity, simultaneously.

## Case report

A 14-year-old Afghan boy was admitted to our hospital, presenting with right shoulder and left thigh pain with prior history of moderate trauma. The pain had gradual onset, increasing over the last 8 weeks.

Physical examination during this visit revealed a febrile patient with oral temperature of 38°C and other vital signs normal. Range of motion of the left ankle was normal but was reduced in the right shoulder. Left ankle, left fibula, and right humerus were tender, and swelling existed in lateral side of the left ankle.

Serological tests showed mild leukocytosis (white blood cell, WBC = 12,000 cells/mL) accompanied with mild polymorphonuclear predominance (70%), elevated alkaline phosphatase (350 IU/L), increased erythrocyte sedimentation rate (119 mm/h), and negative CRP. Results of serum biochemical profile including liver and renal function tests were within normal values.

**Fig. 1:**
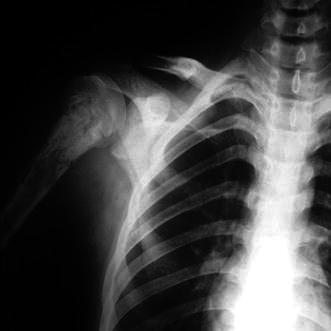
Radiograph showing mixed density, periosteal reaction, and cortical disruption with soft tissue swelling in metaphysis of right humerus

**Fig. 2:**
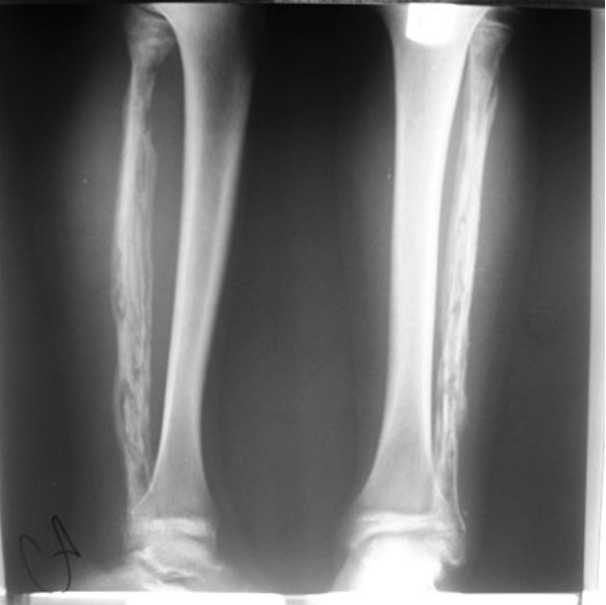
Radiograph showing mixed density, periosteal reaction, and cortical disruption with soft tissue swelling in metaphysis of left fibula

**Fig. 3:**
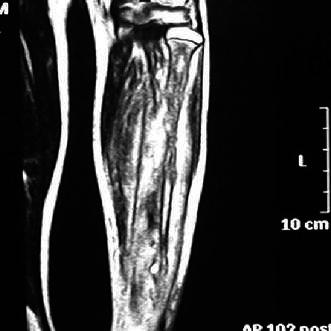
MRI of fibula with signal abnormalities in medulla, metaphysis, and diaphysis

X-ray imaging identified mixed density, periosteal reaction, and cortical disruption with soft tissue swelling in metaphysis of right humerus and left fibula that primarily represented bone malignancy and osteomyelitis (Figs. 1, 2). Computed tomography scan of right humerus and left fibula detected two lesions involving medulla and cortex in head of humerus and body of fibula. In addition, periosteal reaction with soft tissue component and bone marrow infiltration was identified at both sites. On magnetic resonance imaging (MRI), signal abnormalities were found in medulla, metaphysis, and diaphysis of the left fibula, associated with cortical irregularity and diffuse hypersignal areas of soft tissue (Fig. 3). Tibia and knee were normal. Staging of the patient was completed with a whole-body bone scan on which only involvement of right humerus and left fibula was reported. There was no significant inflammation in other parts of the body. Consideration of the radiological and clinical manifestations was useful in development of appropriate differential diagnoses such as metastasis originated from carcinoma (renal cell carcinoma, neuroblastoma), Langerhans cell histiocytosis, and osteomyelitis.

**Fig. 4:**
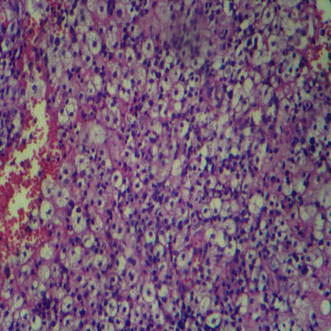
Pathology showing diffuse inflammatory infiltration containing neutrophils and lymph-plasma cells admixed with foamy macrophages

To rule out differential diagnoses, bone biopsy of proximal humerus was done. Grossly, the specimen was soft and yellowish. Microscopically, diffuse inflammatory infiltration containing neutrophils and lymph-plasma cells admixed with foamy macrophages was reported, consistent with xanthogranulomatous osteomyelitis (Fig. 4). Furthermore, a deep fluid sample was analyzed for microbiologic survey. According to histopathology, the patient was started on empiric cloxacillin. Afterwards, cultures exhibited growth of *Staphylococcus aureus* sensitive to cloxacillin. During hospitalization, the patient’s general condition improved. Erythrocyte sedimentation rate measurement was decreased to 35 mm/h, the pain resolved, and he was able to walk. There was no discharge from the site of biopsy, and shoulder range of motion improved. Unfortunately, he left hospital abruptly without surgical debridement or completing the duration of treatment. There was no relapse of pain or discharge at his follow-up appointment within 4 months in an outpatient clinical setting. The patient was informed that data concerning the case would be submitted for publication, and he consented prior to being included in the study.

## Discussion

In the last few decades, Cozzutto reported the first two cases of XO, involving first rib and epiphysis of tibia, respectively [4]. These pseudotumoral lesions are benign in nature, but should be differentiated from malignant disease. In its imaging and clinical manifestations, XO is very similar to carcinoma, but a characteristic histopathological finding can differentiate XO from carcinoma. A correlation between xanthogranulomatous disorders and trauma or infection is hypothetical. As reported in this review, our case had a history of trauma prior to any manifestation. Vankalakunti et al. [5] reported XO of ulna in a 50-year-old postmenopausal woman presenting with 2-year history of progressive swelling in the extensor side of her right forearm. The lesion was curetted out, and cancellous iliac crest graft interposed. Although no organism was found in the tissue culture in that case, *Staphylococcus aureus* was revealed in our patient’s culture.

Cennimo et al. [6] reported a xanthogranulomatous reaction in index finger and wrist of a man complaining of pain and swelling for 1 year, unresponsive to antibiotics. Xanthogranulomatous reaction and positive culture of *Mycobacterium marinum* were demonstrated in his bone biopsy. They performed radical synovectomy of the lesion, administering minocycline, clarithromycin, and ethambutol. A relationship between bacterial infection and xanthogranulomatous inflammation has been determined in several organs such as kidneys and the gastrointestinal (GI) system, but remains undetermined for bone [4, 7–11].

Initially, relying on radiological and gross examination, the list of rare differential diagnoses includes Langerhans cell histiocytoses, Erdheim–Chester disease (ECD), chronic recurrent multifocal osteomyelitis (CRMO), xanthoma, infiltrative storage disorder, malakoplakia, fibrohistiocytic tumor, and metastatic renal cell carcinoma [12–16].

ECD is a rare non-Langerhans cell histiocytosis of unknown etiology, being a multisystemic xanthogranulomatous infiltration with almost constant bone involvement. In our patient, whole-body bone scan failed to show any other site of inflammation, thus ECD was ruled out.

Langerhans cell histiocytosis is a group of idiopathic disorders characterized by proliferation of specialized, bone marrow–derived Langerhans cells and mature eosinophils. Unifocal Langerhans cell histiocytosis presents as a single osteolytic lesion, usually affecting long or flat bones. Multifocal Langerhans cell histiocytosis shows osteolytic lesions involving the calvaria, the sella turcica, the mandible, the vertebrae, and/or the long bones of the upper extremities. Although lesions typically appear granulomatous, with a reactive background of macrophages, eosinophils, multinucleated giant cells, and T cells, the key to diagnosis is identification of pathologic Langerhans cells. The Birbeck granule is their distinctive ultrastructural hallmark [17, 18]. In our case, no Birbeck granules were demonstrated, therefore Langerhans cell histiocytosis was ruled out.

CRMO is a rare condition in which a child’s bones become inflamed and painful. The symptoms are very similar to those of osteomyelitis: unifocal or multifocal, initially osteolytic, later hyperostotic and sclerotic lesions mainly in the metaphyses of the long bones and shoulder girdle, although any bone can be affected [19, 20]. This differential diagnosis was also ruled out histopathologically. Other differential diagnosis was ruled out with the help of biopsy. There is little experience of XO, and we should manage these patients in the light of xanthogranulomatous inflammation in other organs.

We present this case primarily due to its rarity and curability. While xanthogranulomatous osteomyelitis is benign, it can mimic malignant bone lesion in its imaging and clinical manifestations, and appropriate differentiation is crucial. Currently, histopathological examination of lesions is the most specific for diagnosis.
